# A highly sensitive and selective thiosemicarbazone chemosensor for detection of Co^2+^ in aqueous environments using RSM and TD/DFT approaches

**DOI:** 10.1038/s41598-021-00264-z

**Published:** 2021-10-25

**Authors:** Hakimah Ismail, Mohammad Norazmi Ahmad, Erna Normaya

**Affiliations:** 1 Experimental and Theoretical Research Laboratory, Department of Chemistry, Kulliyyah of Science, International Islamic University Malaysia, Jalan Sultan Haji Ahmad Shah, Bandar Indera Mahkota, 25200 Kuantan, Pahang Malaysia; 2 River of Life Kuantan Chapter, International Islamic University Malaysia, Jalan Sultan Haji Ahmad Shah, Bandar Indera Mahkota, 25200 Kuantan, Pahang Malaysia; 3 Innovative Toyyib Environmental Minds, International Islamic University Malaysia, Jalan Sultan Haji Ahmad Shah, Bandar Indera Mahkota, 25200 Kuantan, Pahang Malaysia; 4 Drug and Poison Call Centre, IIUM Poison Centre, Office of Campus Director, International Islamic University Malaysia, Jalan Sultan Haji Ahmad Shah, Bandar Indera Mahkota, 25200 Kuantan, Pahang Malaysia

**Keywords:** Environmental sciences, Analytical chemistry

## Abstract

Chemosensor using organic based compound offering superior alternative method in recognizing metal ion in environmental water. The optimization process strongly affected the performance of the designed sensor. In this study, a highly sensitive and selective colorimetric sensor system utilizing an organic compound, namely thiosemicarbazone-linked acetylpyrazine (TLA), to recognize Co^2+^ ions in different environmental water samples was successfully developed using the response surface methodology (RSM) approach. The developed model was optimized successfully and had statistically significant independent variables (p < 0.05), with optimum recognition occurring in 8:2 v/v DMSO/water at a pH of 5.3, a 100:70 µM TLA/Co^2+^ concentration, and 15 min of reaction time. Under optimum conditions, the TLA sensor recognized Co^2+^ ions at concentrations as low as 1.637 µM, which is lower than the detection limit of flame atomic absorption spectroscopy (FAAS). Theoretical approaches supported the experimental data as well as characterized and predicted the mechanistic non-covalent interactions of TLA-Co^2+^ within the chemosensing system. Finally, all the positive results produced in this study point to TLA as an alternative and comparable probe for recognizing Co^2+^ pollution in water that is cost effective, movable and easy-to-handle, requires no special training and ecofriendly.

## Introduction

Cobalt ion (Co^2+^) is a trace element that distributed widely in nature. This valuable metal has applications in wide range of industrial processes, such as the production of steel and alloys, rechargeable batteries, nanotechnology products, fertilizers, and medicines. Due the high demand, its production and industrial applications subsequently impact the environment. Discharged waste containing Co^2+^ ions may come into contact with the soil, water, rocks and living organisms^1^. It tends to accumulate in living organisms and environmental systems because it is not biodegradable. Human exposure to Co^2+^ ions usually occurs through the dietary intake of contaminated food, such as fish, meat, dairy products and supplements, the treatments of several diseases, environmental pollution and industrial activities^2^. Co^2+^ ions exposure is normally low. However, excessive exposure to Co^2+^ ions may lead to Co^2+^ toxicity, which can affect various biological systems, such as the cardiovascular, nervous, endocrine, and respiratory systems^2^.

Therefore, a suitable and fast technique is needed to detect the presence of Co^2+^ ions in environmental water. Many techniques are available for Co^2+^ detection, including flame atomic absorption spectroscopy (FAAS), graphite furnace atomic absorption (GFAA), atomic emission spectroscopy (AES), inductively coupled plasma mass spectrometry (ICP-MS), inductively coupled plasma optical emission spectroscopy (ICP-OES), neutron activation analysis and voltammetry^3^. These techniques are sensitive in terms of sensing metal ions but have several disadvantages. The analyses must be performed by trained personnel in the laboratory, making them time consuming and high cost^3^. Due to their disadvantages and limitations, many studies have sought to use organic-based sensors as alternatives for recognizing various metal ions in water. The technique is user-friendly, is easy to operate, features rapid field analyses, is highly selective and is sensitive towards the selected metal ions. Many studied have been reported for Co^2+^ recognition using an organic-based colorimetric sensor^4–14^. However, most of the sensors only focused on application toward drinking and tap water. Fewer studied on developing a colorimetric sensor for Co^2+^ ions recognition in different environmental water have been developed and reported. In this study, one of the organic-based colorimetric sensors, namely thiosemicarbazone-linked acetylpyrazine (TLA), has used against Co^2+^ ions. To produce an organic-based sensor with high sensitivity and selectivity towards the targeted metal ions in different environmental water samples, an optimization study must be performed.

Previously, the optimizations of Co^2+^ sensors were done using the conventional method^4–14^, which uses a one-variable-at-a-time technique that varies one variable at a time while keeping the other parameters constant and observing the effect on the yield or response. Using this method, it is possible that interactions between variables may not be observed and examined properly, perhaps resulting misinterpretations of the result^15^. It also may increase the number of experiments necessary and the overall cost. Therefore, to overcome these problems, optimization studies can be done using the response surface methodology (RSM) approach, which is a statistical and mathematical analysis method that can analyse the comprehensive effect of several variables on the response of interest^15^. The other advantages of this method are that it is able to reduce the number of runs and costs^15^. The method is also capable to study and visualize the interactions among the variables in a three-dimensional plot^15^. These results are crucial to study overall response in sensing system, specifically in improving its sensitivity and selectivity towards targeted metal ions in different environment water samples. This study also used different theoretical approach and technique from the previous developed Co^2+^ sensor^4–14^ in visualizing and clarifying the mechanistic interaction occurring between the developed sensor and Co^2+^, which are density functional theory/time-dependent density functional theory (DFT/TD-DFT) and non-covalent interaction-reduced density gradient (NCI-RDG) methods. The practicality of using TLA to recognize Co^2+^ ions in different environmental water samples are also investigated using UV–Vis and, cellulose-based test strips analysis.

## Results and discussion

### Statistical analysis and modelling using RSM

The optimum condition under which TLA recognizes Co^2+^ ions was statistically identified using the RSM approach. To determine the optimum absorbance at the center point, 20 experiments were designated for the three-factor face-centred analyses, as shown in Supplementary Table S1. The results show that the absorbance range of TLA for recognizing Co^2+^ ions when using the modelled ranges of the variables runs from 0.1691 to 0.3501. The lowest and highest absorbance were obtained for runs 7 and 13, where the variables were set to the following values for the concentration of Co^2+^, pH and reaction time, respectively: 40 µm, 7.5, 20 min and 80 µm, 4.5 and 20 min.

The three variables were successfully investigated via the absorbance values they generated using UV–Vis spectroscopy, and their predicted absorbance values were found via a multiple regression analysis, which produced the Eq. (1) below.

1 $$ \begin{aligned} {\text{Absorbance }} = & \, - {3}.{323 } \times { 1}0^{{ - {1}}} + { 7}.{516 } \times { 1}0^{{ - {3}}} *A + { 1}.{635} \times { 1}0^{{ - {1}}} *B \\ & - { 2}.{684 } \times { 1}0^{{ - {3}}} *C + { 9}.000 \, \times { 1}0^{{ - {5}}} *AB + { 3}.{725 } \times { 1}0^{{ - {5}}} *AC \\ & - { 4}.0{33 } \times { 1}0^{{ - {4}}} *BC - {5}.{56}0 \, \times { 1}0^{{ - {5}}} *A^{{2}} - { 1}.{6}0{6 } \times { 1}0^{{ - {2}}} *B^{{2}} + { 6}.{236 } \times { 1}0^{{ - {5}}} *C^{{2}} \\ \end{aligned} $$

where *A*, *B* and *C* are the Co^2+^ concentration, pH value and reaction time, respectively; *AB*, *AC* and *BC* are the interaction terms; and *A*^2^, *B*^2^ and *C*^2^ are the quadratic terms for these variables.

The significance and adequacy of RSM model used in this study to optimize the sensitivity of TLA towards Co^2+^ ions was determined by performing the analysis of variance (ANOVA) shown in Supplementary Table S2. The model produced a p-value of less than 0.05, and the lack of fit is more than 0.05. These two results suggest that the model fitness is significance (p < 0.0001) and that the lack of fit is insignificance, indicating that all the variables used were suitable and well-fitted in terms of the experimental data^16^. In addition, the probability of noise in this model is low^17^.

Supplementary Table S3 shows the fit statistics for the model. The low coefficient of variation (CV) and low prediction residual error sum of squares (PRESS) at 0.9396 and 0.0006, respectively, indicate that the model in this study is precise and reliable^17^. The aptness of the model is also evidenced by a multiple correlation coefficient (R^2^) of 0.9984, which is close to 1.00. The values of the predicted R^2^ and adjusted R^2^ are very close at 0.9887 and 0.9970, respectively, showing that they are in a reasonable agreement and indicating a highly significant model^18^. When expressed as a percentage, the predicted R^2^ of 98.87% indicates that 98.87% of the variability in the response variable can be explained by this proposed model. The adequate precision value, which is a measure of the signal-to-noise ratio, also has a value greater than 4 at 91.8596, indicating the model is adequate and a good fit^17^.

### Response surface and contour plot for interaction analysis

After successfully identifying the type of model formed and checking its adequacy, the interactions between variables that contribute to the optimum response in this study were analysed based on the response surface and contour plots. In this study, the central composite design (CCD) was used to optimize the independent variable at three different levels (high, medium, low), and response surface graphs were plotted based on the effects of the variables on the absorbance values for TLA recognizing Co^2+^ ions. The graphs were obtained by keeping one of variable constant while varying the other two variables and predicting the response.

**Figure 1:**
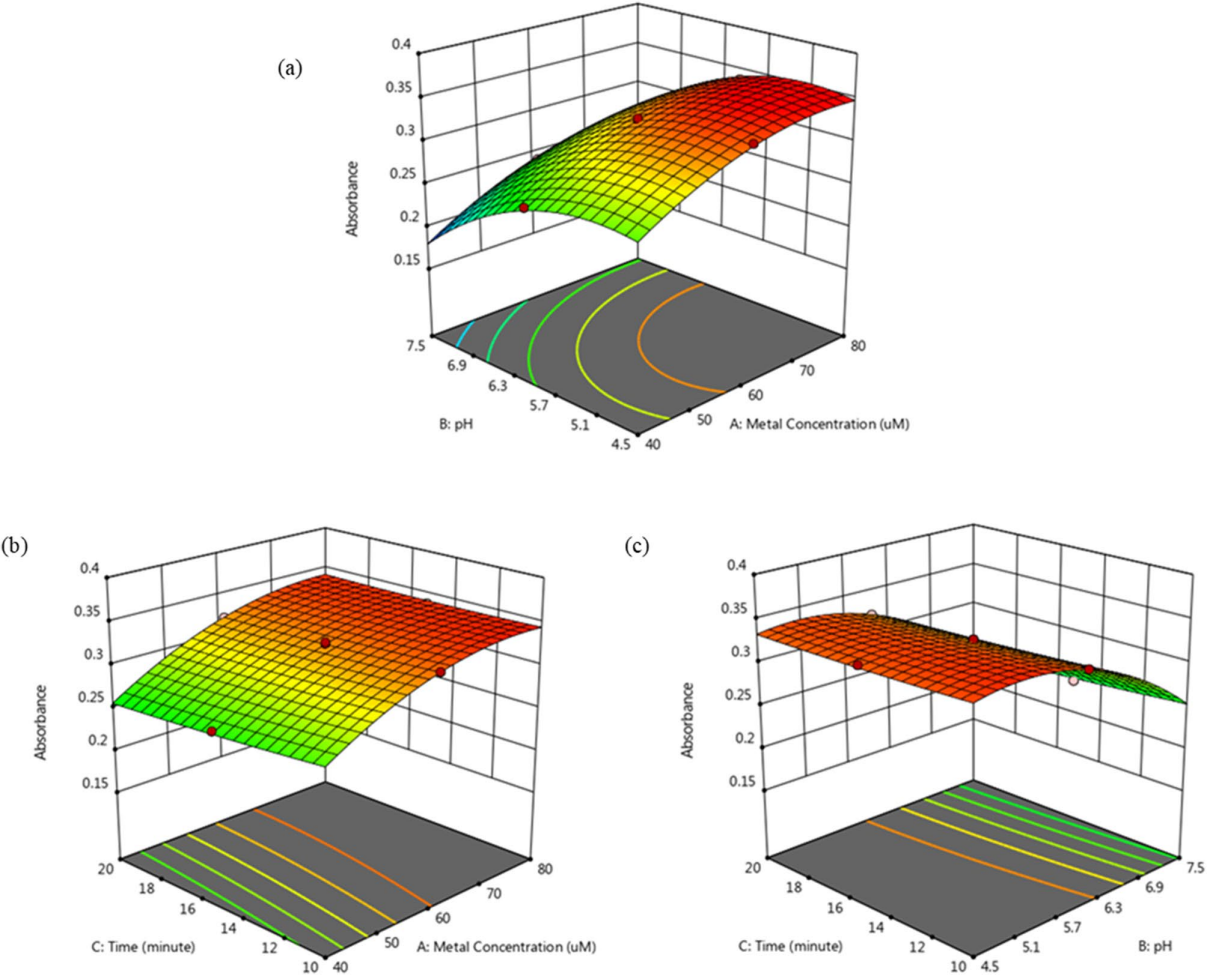
Three-dimensional (3D) plots for the absorbance of TLA-Co^2+^ versus (**a**) Co^2+^ concentration and pH, (**b**) Co^2+^ concentration and reaction time and (**c**) pH and reaction time.

The interactions between variables can be categorized as significant or insignificant in this study based the plot shapes and p-values. Figure 1 shows that all the contour plots have elliptical shapes, which means that the interactions between the variables studied (concentration of metal ions (A), pH (B) and reaction time (C)) are significant in terms of the response variable (absorbance (Y)), which is supported by all the p-values for the interactions (AB, AC and BC) being less than 0.05^18^. Figure 1a shows the effect of the pH and metal concentration on TLA recognizing Co^2+^. The result shows that the higher absorbance (response) of TLA in recognizing Co^2+^ can be achieved for concentrations within 60–80 µM and pH values in the 4.5–6.3 range. Figure 1b,c also show that the reaction time contributes its effect to the absorbance value, which is supported by p-values for its interactions of p < 0.0036 and p < 0.0118, respectively. The three variables have significant effects on the response in terms of optimizing TLA as a chemosensor in recognizing the Co^2+^ ion. According to the t-values, the pH had the strongest effect on the response in this study, followed by metal ions concentration and reaction time, where the t-values were 50.91, 44.12 and 5.67, respectively. Using this RSM model, the optimum condition in terms of TLA recognizing Co^2+^ has been successfully identified as occurring in 8:2 v/v DMSO/pH 5.3 with a 15-min reaction time, where the concentration of Co^2+^ is 70 µM in 100 µM of TLA.

### Method validation

A validation test for the optimum conditions under which TLA recognizes Co^2+^ was performed in triplicate, where the parameters followed the optimum conditions, and the absorbance values were compared with the predicted values from Eq. (1). The triplicate tests show that the optimum response successfully achieved an average absorbance value of 0.3484 ± 0.0131 using the model Eq. (1). Thus, there is a 99.25% agreement between the experimental and predicted values. The results are also supported by Tukey test, which indicates that, with 99% certainty, there is no significance difference between the experimental and predicted values.

### Sensitivity and selectivity of the chemosensor

**Figure 2:**
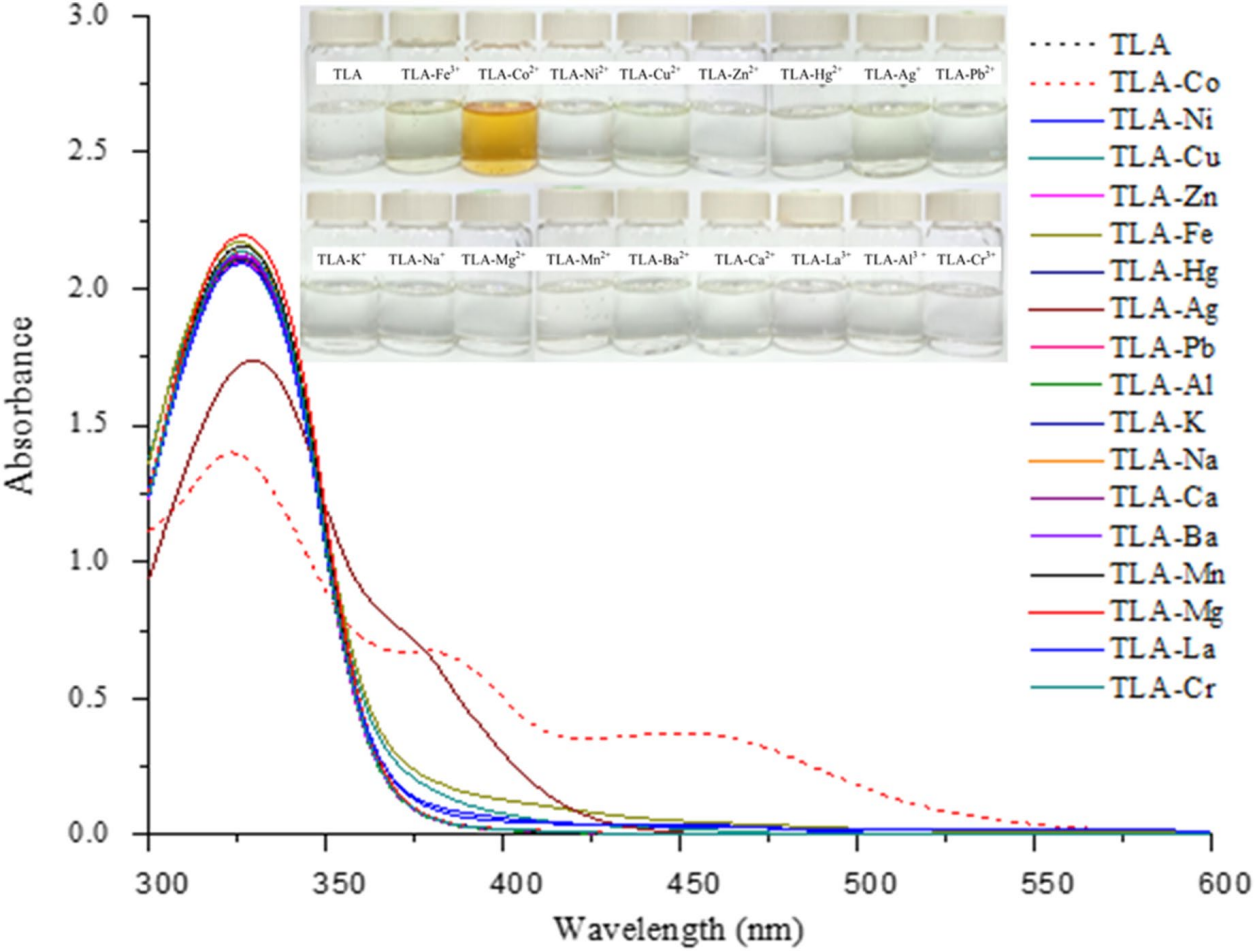
Absorption spectra of the TLA chemosensor under the optimum condition (100 μM of TLA in 8:2 v/v DMSO/pH 5.3) towards various metal ions and its color of the TLA complexes formed with various metal ions.

After finding an adequate model for the optimal condition under which TLA recognizes Co^2+^ ions, TLA’s sensitivity and selectivity is investigated and validated. The selectivity of TLA towards 17 types of metal ions is investigated under these optimum conditions. Figure 2 shows that a significant interaction takes place after TLA recognizes Co^2+^ in an aqueous medium compared to what happens with the other metal ions. It shows that under this optimal condition from the RSM model, the other 16 metal ions did not have any significant interactions with the TLA chemosensor. These results can also be observed with the naked eye by showing that the interaction between TLA and Co^2+^ also produces significant colour compared to the interactions with the other metal ions. It produces as orange colour, as can be seen with the naked eye, while the other interactions took place in colourless solutions. The formation of new bands at 383 and 450 nm, together with orange color of the solution, correspond to the color wheel theory that the TLA chemosensor interacted with the Co^2+^ in a selective fashion. The predicted model of interaction between TLA and Co^2+^ and its electronic transitions will be discussed in detail in the theoretical sections.

### Interference analysis

**Figure 3:**
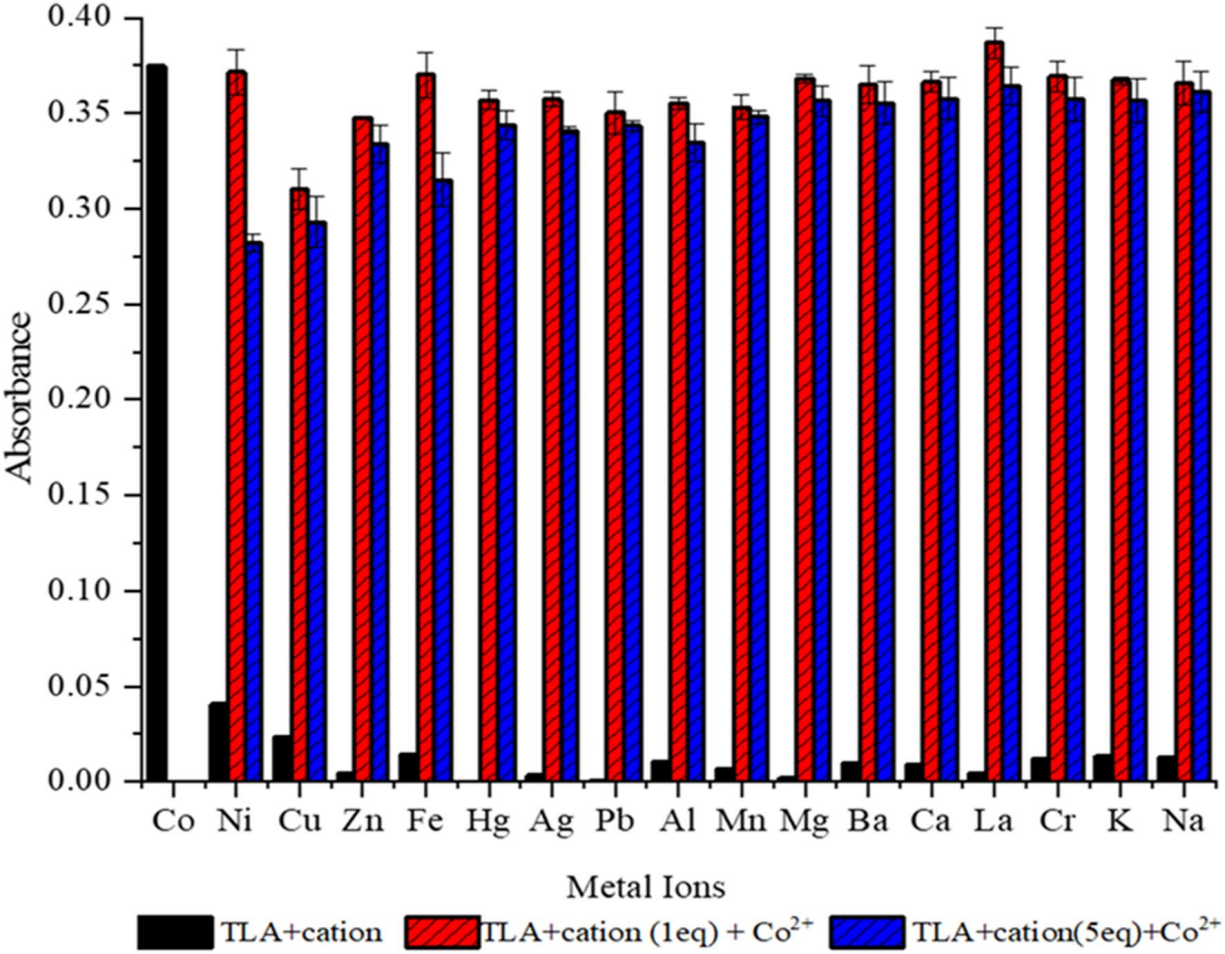
TLA selectivity against Co^2+^ with different equivalents of other cations.

The selectivity of TLA chemosensor in recognizing Co^2+^ in the presence of other metal ions was further investigated through an interference analysis. As shown in Fig. 3, the co-existence of other metal ions in different equivalences (1 and 5) did not have any significant effect on the absorbance value of TLA-Co^2+^, thus proving that TLA has higher selectivity in recognizing Co^2+^ even in the presence of other 16 metal ions.

### Limit of detections (LOD)

**Figure 4:**
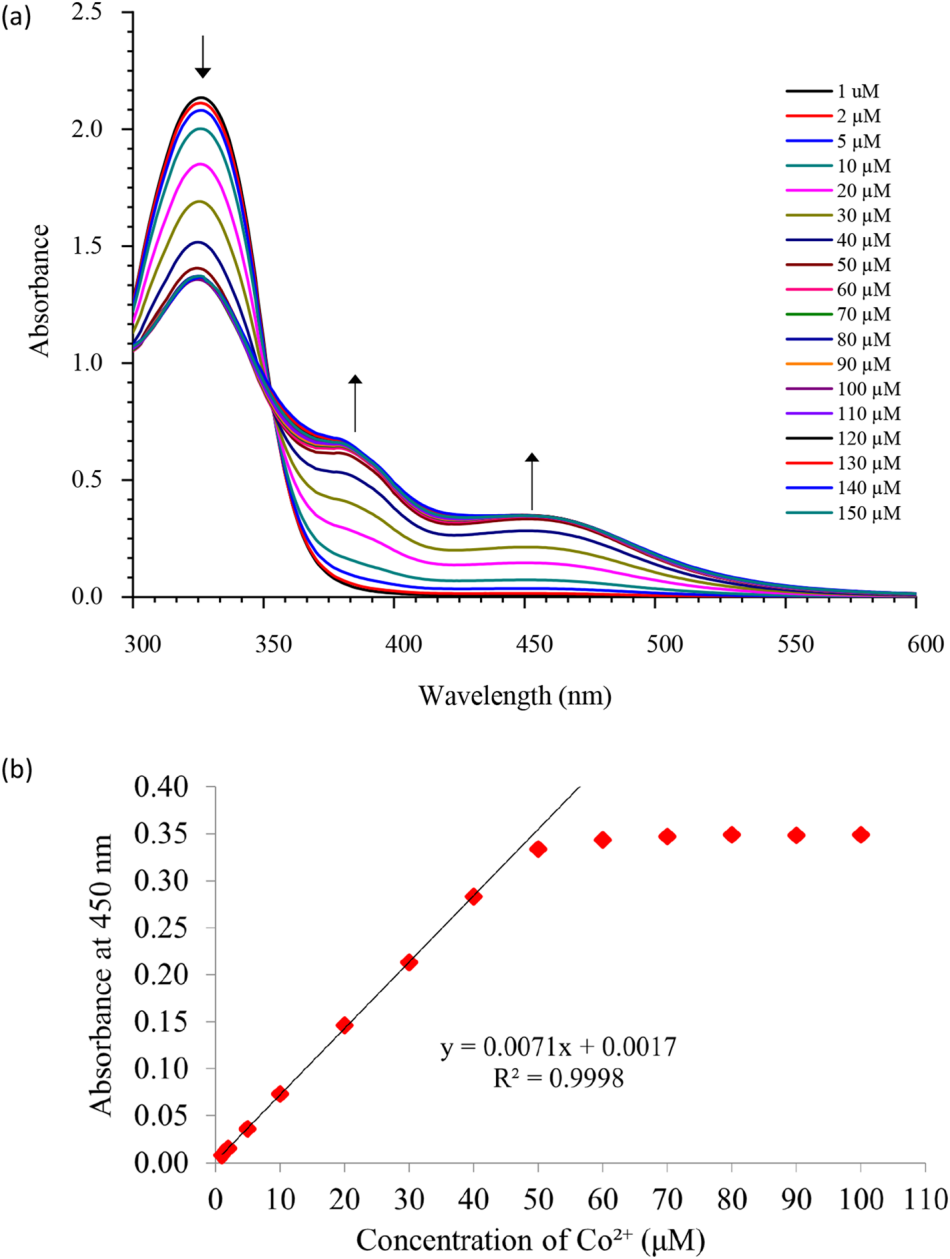
(**a**) Sensitivity plot of TLA-Co^2+^ for 1–150 μM of Co^2+^ under the optimum condition (100 μM of TLA in 8:2 v/v DMSO/pH 5.3) and (**b**) calibration curve for absorbance at 450 nm against Co^2+^ concentration.

Figure 4 shows the sensitivity plot of TLA-Co^2+^ for 1–150 µM of Co^2+^ under the optimum condition using the titration method. The gradual increment of Co^2+^ from 1 to 50 µM caused the reduction of the absorption intensity of the complex at 328 nm, while the absorbance intensity increased at 383 and 450 nm, respectively. The absorbance values were constant from 50 to 100 µM, as the interaction reached the maximum due to the limited TLA molecules. An isosbestic point was clearly identified at 354 nm, indicating the complete transition of TLA to TLA-Co^2+^, and subsequently showed that only one product was produced in the system^6,10,12^.

The LOD was determined with the IUPAC equation (C_DL_ = 3Q/S), where Q is the standard deviation for the blank solution, and S is the slope of the calibration curve^19^. Ten replications of the blank solution were used for the standard deviation. The calculated results showed that the lowest concentration of Co^2+^ which can be detected by the TLA sensor using UV–Vis analysis is 1.637 µM. The result shows that the TLA sensor is comparable and could be an alternate probe to recognize the presence of Co^2+^ ions as compared with the previously studied as shown in Table 1^4–14^. The factors that contributed to the high sensitivity of the TLA sensor will further discussed in theoretical section.

**Table 1:** Comparison of previous reported organic-based sensors/probes for Co^2+^ ions recognitions.

Organic-based sensor	LOD	Application on water sample		References
		UV–Vis	Test strip	
(E)-1-(2-((1H-imidazol-2-yl) methylene) hydrazinyl)phthalazine	65 nM	Drinking, tap water	Test strip to different concentrations of Co^2+^ and various metal ions	^4^
Schiff base with 2-hydrazinyl-4-(trifluoromethyl)pyrimidine and pyridine moieties	0.11 μM	Drinking, tap water	Test kit to various metal ions in buffer solution	^5^
A mixture of 2,2’-dihydroxyazobenzene and 2,2’:6’,2’’-terpyridine	0.45 μM	–	–	^6^
Combination of 4-diethylaminosalicylaldehyde and diethylenetriamine	0.65 μM	Drinking, tap water	Test strip to prove Co^2+^ and various metal ions	^7^
(6,6′-((1E,1′E)-((thiobis(2,1-phenylene)) bis(azanylylidene)) bis(methanylylidene)) bis(2-methoxyphenol))	0.66 μM	Drinking, tap water	–	^8^
N 2-(bis(pyridin-2-ylmethyl) amino)-*N*-(2-((2,4-dinitrophenyl) amino) phenyl) acetamide	0.99 μM	Drinking, tap water	–	^9^
(1 = 2-(N-(2-hydroxybenzyl)-N-((pyridin-2-yl) methyl) amino)-N-(2-hydroxyphenyl) acetamide)	1.8 μM	Drinking, tap water	–	^10^
2-(5-(2-formylphenyl)-1,3,4-oxadiazol-2-yl) benzyl picolinate	3.92 μM	Drinking, tap water	Test paper to prove Co^2+^ in water	^11^
Comprises of quinoline and N^1^, N^1^-dimethylethane-1,2-diamine	6.89 μM	Drinking, tap water	–	^12^
Coumarin based azomethine	7.09 μM	Distilled, drinking, ditch, industrial, lake, sea, river water	–	^13^
A mixture of methylene blue, 2-aminothiophenol and copper nitrate	0.04 mM	–	–	^14^
The proposed sensor (TLA sensor)	1.637 μM	Distilled, tap, lake, river, mangrove, sea	Test strip to various concentrations of Co^2+^ in environmental water samples	–

### Job’s plot

The stoichiometry of the interaction that occurs between TLA and Co^2+^ was also studied using the Job’s plot method. The mole fraction of Co^2+^ was varied from 0 to 0.9 in a solution of Co^2+^ and TLA to obtain the Job’s plot data. The graph of the absorbance versus the mole fraction at a wavelength of 450 nm is plotted in Supplementary Fig. S1. The results indicate that a 2:1 stoichiometry ratio of TLA to Co^2+^ is used to recognize Co^2+^ in the chemosensing system.

### Conductor-like screening model for realistic solvents (COSMO-RS)

The molecular polarization, or interaction, of TLA in its medium was studied theoretically using the COSMO-RS approach. The probability distribution of the molecular surface segment having a specific charge density was present as a sigma profile versus the screening charge density, as shown in Supplementary Fig. S2. There are three regions present in this graph representing a H-bond donor region (> − 0.0084 e/A^2^), non-polar region (− 0.0084 to 0.0084 e/A^2^) and H-bond acceptor region (< 0.0084 e/A^2^)^20^. Peaks appearing at greater positive and negative values of screening charge densities of DMSO and TLA suggest that DMSO is the best medium for TLA in terms of acting as a chemosensor via the formation of a hydrogen bond^21^. A hydrogen bond interaction between the chemosensor and it medium is crucial in terms of increasing its sensitivity towards targeted metal ions. This interaction contributes a smaller energy gap between the ground and excited states, and it corresponds to the increase in the reactivity (sensitivity) of the chemosensor towards the targeted analyte. The process occurs due to the destabilization energy at the ground state position^20^.

### Fukui function

**Figure 5:**
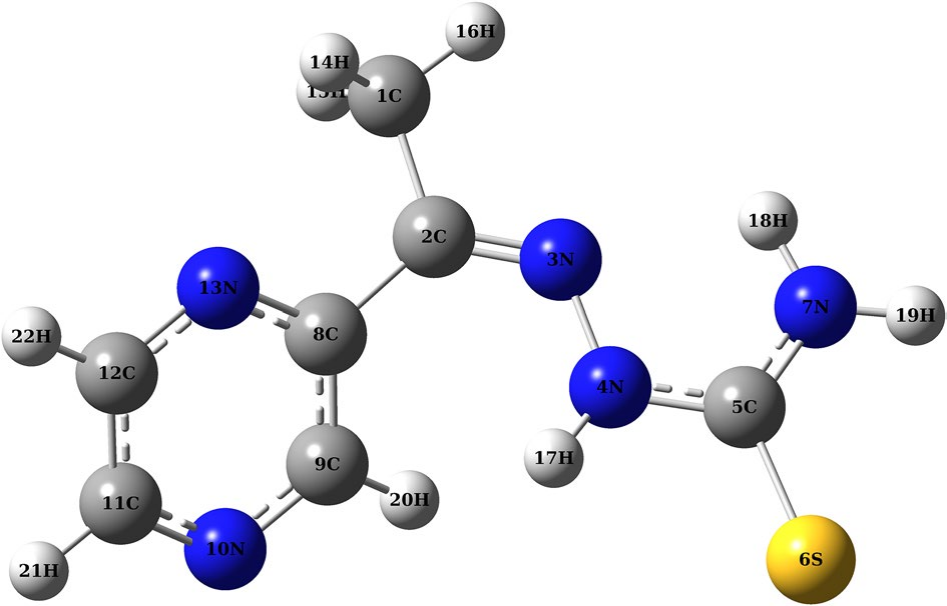
Optimized TLA structure.

The correlation coefficient between experimental (Pub Chem CID = 9574643) and optimized TLA structure (Fig. 5) was plotted, as shown in Supplementary Fig. S3. The results indicated reliable agreement between the experimental and theoretical values before further calculations were performed. The Fukui Function approach was calculated to identify the specific atoms prone to be in the nucleophilic and electrophilic regions in this study. The character of each specific atom was calculated using the Eq. (2) below^22^:

2 $$ \begin{aligned} & f_{{\text{k}}}^{ + } = \, \left[ {q\left( {N + { 1}} \right) \, {-}q\left( N \right)} \right]{\text{ for a nucleophilic attack}}, \\ & f_{{\text{k}}}^{-} = \, \left[ {q\left( N \right) \, {-}q\left( {N{-}{ 1}} \right)} \right]{\text{ for a electrophilic attack}}, \\ & f_{{\text{k}}}^{0} = { 1}/{2}\left[ {q\left( {N + { 1}} \right) \, {-}q\left( {N{-}{ 1}} \right)} \right]{\text{ for a radical attack}}, \\ \end{aligned} $$

where *N*, *N* − 1 and *N* + 1 are the total electrons present in the neutral, cationic and anionic states of the molecule, respectively. The equation from *f*_*k*_^*−*^ was used to identify the atom most susceptible to electrophilic attack. As shown in Supplementary Table S4, the possible binding site of TLA for the detection of Co^2+^ were identified as atom S(6) and N(10) which had the highest electron density values of 0.7304950 and 0.161610, respectively.

### Time-dependent density functional theory (TD-DFT)

**Figure 6:**
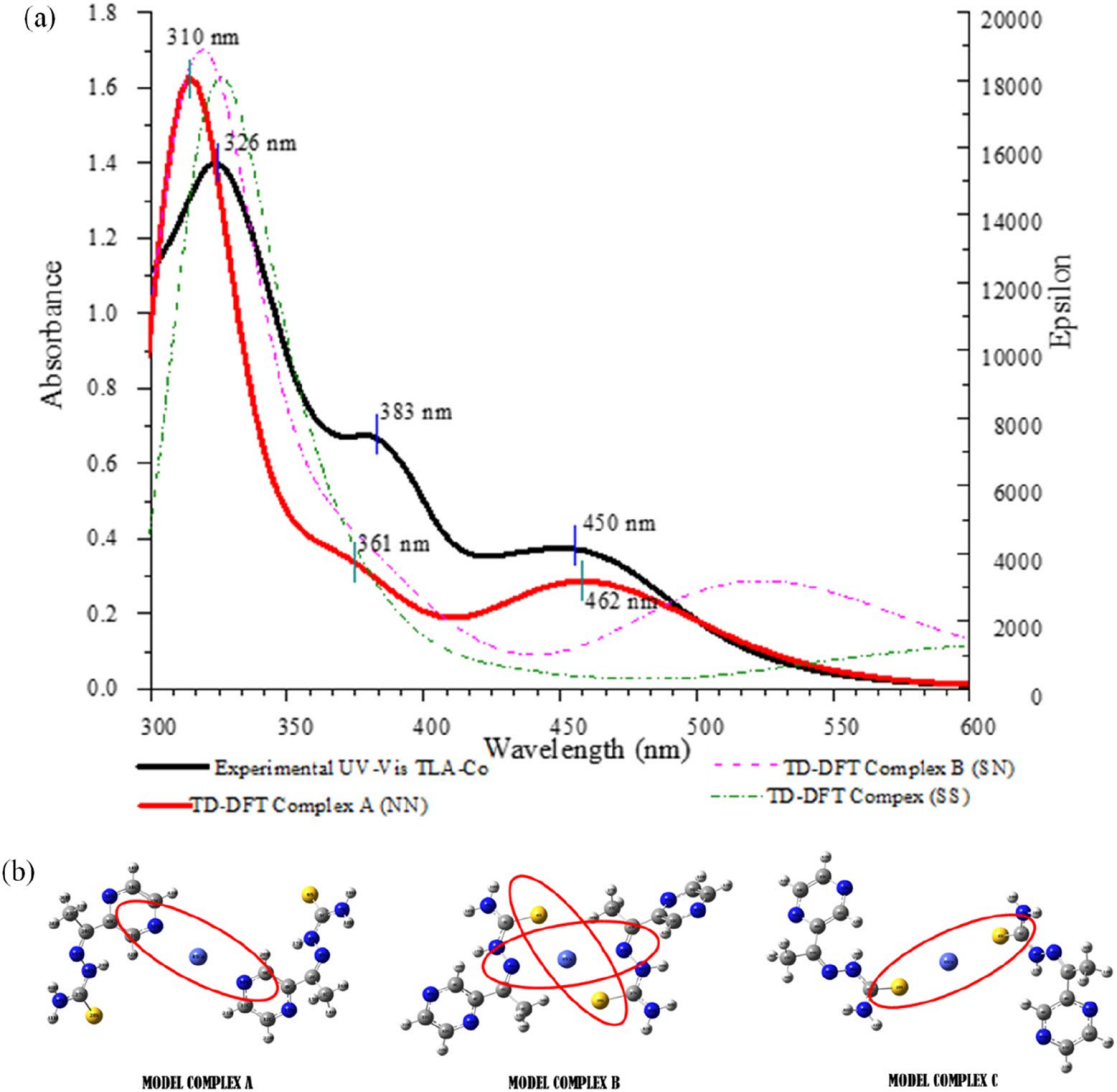
(**a**) The experimental and calculated UV–Vis spectra for the TLA–Co^2+^ complexes (**b**) model for TLA-Co^2+^ complexes.

The TD-DFT method was used to validate the suggested structure of complex and interaction between TLA and Co^2+^ ions. The designation of the suggested complexes is based on the Fukui function and Job’s plot results. Experimental spectrum of TLA-Co^2+^ will be used as a control in identifying the corresponding structure of complex and interaction formed. As shown in Fig. 6, significant differences were identified for complexes B and C based on spectrum shapes and absorbance wavelengths. Complex A exhibited the most identical spectrum shapes and absorbance wavelengths between the experimental and TD-DFT calculation, indicating it was the suggested of the complex structure, and N atom from pyrazine ring is the site of interaction occurred for TLA in recognizing Co^2+^ ion in this chemosensing system.

The suggested structure (complex A) in the chemosensing system aligns with the hard and soft acids and bases (HSAB) principle. N atom from TLA structure shows that it is the preferable site to interact against Co^2+^ due to its moderate base and acid properties, respectively. The suggested model also showed that the position of the N atom from the pyrazine has a small steric effect, thus increasing the reaction rate of the reaction to occur in the system. These factors contributed significantly to the high sensitivity and selectivity of TLA in recognizing Co^2+^ as lowest as 1.637 µM, comparing other developed sensors^10–14^.

The type of electronic transitions and their assignments occurred in the system was further characterized using the suggested model. As shown in Supplementary Table S5, the characterization is based on a contour plot overview (Supplementary Fig. S4) and the relative energies of the electronic transitions between the localized and delocalized molecular orbitals involved. Three bands were observed at absorbance wavelengths of 326, 383 and 450 nm for under the experimental conditions and at 310, 362 and 457 nm for the TD-DFT method, respectively. Two types of electronic transitions occurred at 310 nm, namely the n − π* and π − π* electronic transitions. These two characters occurred without involving the interaction between the TLA and Co^2+^ ions, and the transitions were from HOMO-11 towards LUMO, HOMO-11 towards LUMO + 4 and HOMO-7 towards LUMO + 4, with contributions of 35, 28 and 26%, respectively. The metal ligand charge transfer (MLCT) electronic transition occurred at a calculated peak 362 nm, with a 91% contribution from HOMO-3 towards LUMO + 1. The calculated peak at 457 nm were for the MLCT and ligand metal charge transfer (LMCT) that occurred between TLA and Co^2+^ from HOMO towards LUMO + 7. The occurrence of these two peaks in both the experimental and calculated spectra proves that an interaction occurred between TLA and the Co^2+^ ions in the chemosensing system.

### Non-covalent interaction-reduced density gradient (NCI-RDG)

The model suggested by the TD-DFT approach was further utilized to characterize the type of interaction occurring between TLA and Co^2+^ ions in this chemosensing system in conjunction with the NCI-RDG approach. The natures of the intermolecular and intramolecular interactions taking place in the system were identified by plotting the RDG against sign(λ_2_), as shown in Supplementary Fig. S5. Three types of interaction can be present based the regional values, namely repulsive/non-bonding interaction [(sign(λ_2_) ρ > 0], attractive interaction [(sign(λ_2_) ρ < 0] and van der Waals interaction [(sign(λ_2_) ρ ≈ 0]^23^. Based on the results, two types of interaction occur in the system, i.e., repulsive/non-bonding, and attractive/hydrogen bond interactions. To visualize the locations of these interaction types within the system, the gradient isosurface for the real space of the molecule is shown in Supplementary Fig. S5b. The result shows that the type of intermolecular interaction occurring when TLA recognizes Co^2+^ is a hydrogen bond^24^ and there are four locations of intramolecular interaction formed in each TLA chemosensor in this chemosensing system.

### Applications in water samples

**Figure 7:**
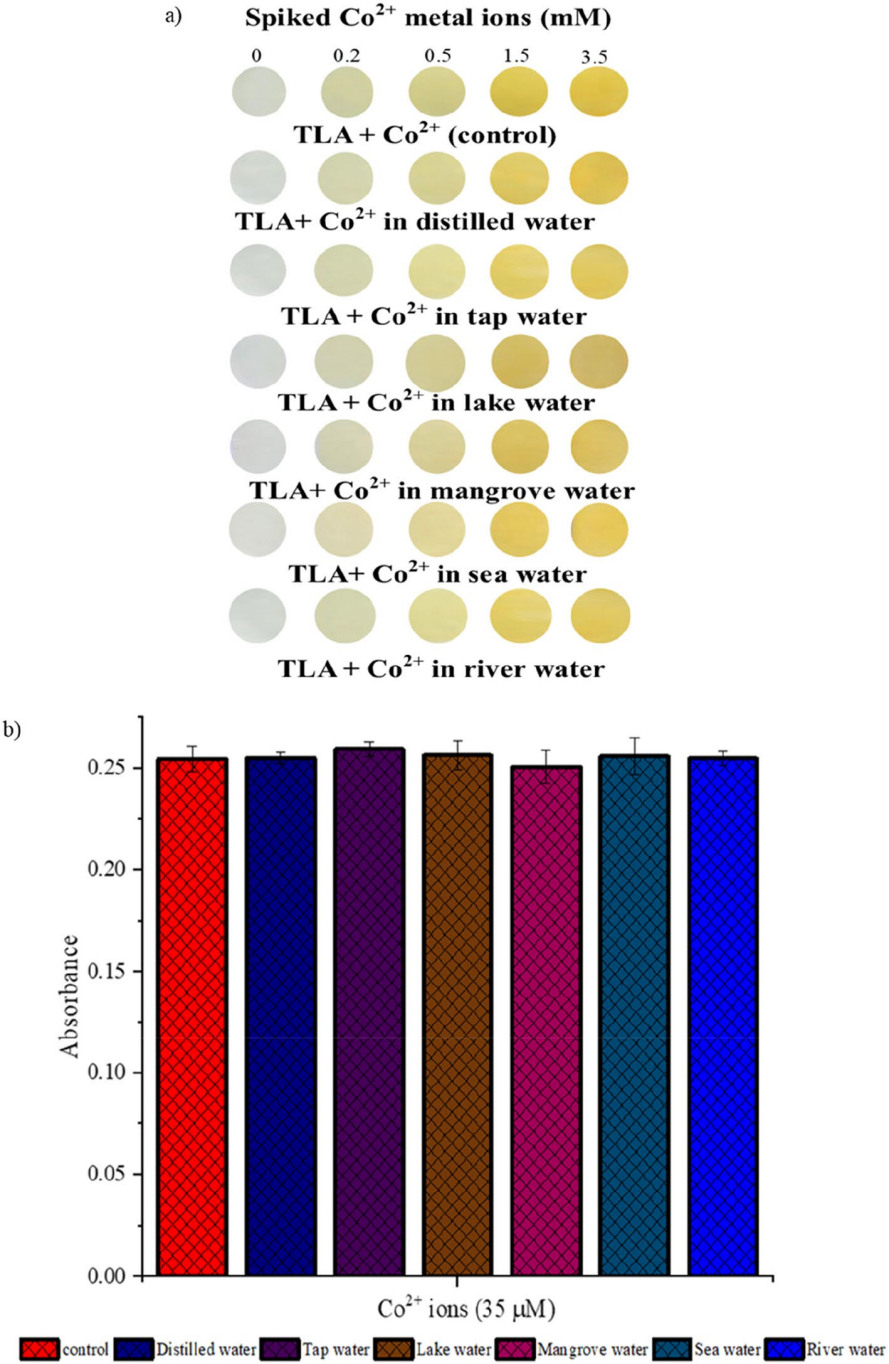
(**a**) Test strips of TLA immersed in spiked water samples (**b**) absorbance at 450 nm of the spiked water samples.

The optimized TLA sensor was tested on five spiked water samples to validate the use of this sensor to test real environmental samples, based on naked-eye observations of test strips and UV–Vis analysis. The orange color increased intensity with increasing concentrations of Co^2+^ ions, as shown in Fig. 7a. The result showed that TLA test strips for Co^2+^ detection appear to be functional and practical, and millimolar concentrations of Co^2+^ ions can be detected with the naked eye. For the UV–Vis analysis, there were no significant differences (p < 0.05) between five spiked water samples, as shown in Fig. 7b. The results were also validated using ICP-MS measurements, as shown in Table 2. Tukey’s test was used to validate both methods, and no significant difference (p < 0.05) was found. The results showed that the TLA formed as a sensitive and selective sensor, and it can be used as an alternative probe for detecting Co^2+^ ions in environmental water samples.

**Table 2:** Determination of Co^2+^ ions in water samples.

Water sample	Spiked (µM)	UV–Vis		ICP-MS	
		Found (µM)	Recovery (%)	Found (µM)	Recovery (%)
Control	35	35.59	101.68	35.53	100.51
Distilled water	35	35.67	101.92	35.06	100.17
Tap water	35	36.27	103.64	35.35	101.00
Lake water	35	35.84	102.39	35.41	101.60
Mangrove water	35	35.05	100.13	35.28	100.80
Sea water	35	35.76	102.17	35.38	101.08
River water	35	35.62	101.77	35.44	101.26

## Conclusion

We have successfully developed the TLA chemosensor with sensitivity and selectivity towards targeted metal ions (Co^2+^) in different environmental water samples using the RSM model. Optimization through the RSM showed that the interaction of TLA and Co^2+^ was optimal at 8:2 v/v DMSO at pH 5.3, a 100:70 μM TLA/Co^2+^ concentration, and a 15-min reaction time. The binding stoichiometry of TLA–Co^2+^ was 2:1. After optimization, TLA’s applicability as a chemosensor for recognizing Co^2+^ ions was easy to monitor and visualize using the naked eye and UV–Vis analysis because the test strips changed from colorless to orange, and redshifts occurred at 383 and 450 nm, respectively. The LOD was calculated to be 1.637 μM, indicating the sensor is comparable and capable of being used for environmental water samples. Using the DFT approach, the N atom from the pyrazine substituent was successfully identified as the most reactive atom in recognizing Co^2+^. The TD-DFT method successfully determined the suggested interaction model between TLA and Co^2+^ ions in the chemosensing system. Using an NCI-RDG calculation, the type of interaction occurring between TLA and Co^2+^ was successfully identified as a hydrogen bond. All the positive results produced in this study point to TLA as an alternative probe with a highly sensitive and selective colorimetric chemosensor for recognizing Co^2+^ pollution in water that is movable and easy-to-handle and requires no special training.

## Material and methods

### Materials and instruments

All chemicals and solvents were purchased from Aldrich, HmBG, QReC, Riendemann, Merck, R&M, ACS, Systerm, Fisher Scientific and UniLab Chemicals. Deionized water was used throughout the experiment. The synthesized TLA was characterized through melting point analysis, FTIR spectroscopy (Frontier, PerkinElmer, USA), CHNS elemental analysis (2400 Series II, PerkinElmer, USA) as well as ^1^H and ^13^C-NMR spectroscopy (Ultra Shield Plus, Bruker, USA). The optimization of TLA as a colorimetric sensor was conducted using UV–Vis single-beam (UVmini-1240, Shimadzu, Japan) and double-beam (Lambda 35, PerkinElmer, USA) spectrophotometers.

### Synthesis and characterization

TLA was synthesized by mixing a 1:1 molar ratio of 2-acetylpyrazine and thiosemicarbazide in boiling ethanol^7^. A few drops of concentrated sulphuric acid (95–98%) were added as a catalyst to shorten the reaction time. The solution was heated to reflux for 2 h, and its reaction is shown in Supplementary Fig. S6. The TLA precipitate formed after cooling was collected via vacuum filtration. Yield: 96%; mp: 223.0–224.5 °C; ^1^H NMR (Fig. S7) (500 MHz, DMSO-*d*_6_, ppm): δ 10.49 (s, 1H), 9.66 (d, *J* = 1.4 Hz, 1H), 8.62–8.60 (m, 2H), 8.49 (s, 1H), 8.33 (s, 1H), 2.37 (s, 3H); ^13^C NMR (Fig. S8) (400 MHz, DMSO-*d*_6_, ppm): δ 179.70, 150.55, 146.69, 144.38, 143.72, 143.51, 12.31; FTIR (Fig. S9) (KBr, cm^−1^): 3370.3 (N–H), 3249.2 (N–H), 3170.7 (N–H), 1617.7 (N–H); analysis (calcd., found for C_7_H_9_N_5_S): C (43.06, 43.18), H (4.65, 4.78), N (35.87, 35.54).

### RSM

To produce colorimetric sensor of TLA that has high sensitivity and selectivity in recognizing Co^2+^ ions, three independent variables (Co^2+^ concentration (40–80 μM), pH (4.5–7.5) and reaction time (10–20 min) were selected for optimization via the RSM model. Design-Expert 11.0 (Stat-Ease Inc., Minneapolis, MN, USA) was used to generate the experimental design and analyse the data with regard to the optimization of TLA-Co^2+^. The optimization was conducted using the face-centred CCD, with its absorbance value at the selected wavelength obtained from the medium screening process. The experimental factors and levels used in the RSM experiment are shown in Supplementary Table S6. Zero was set as the conventional level for each variable. Three different levels, namely low, medium and high, were studied as independent variables. Twenty experiments were set up based on the chosen parameters.

### Statistical analysis and modelling

A second-order polynomial equation was fitted to the data via multiple regression procedures after the completion of the RSM experiments. The Design Expert 11.01 (Stat-Ease, Minneapolis, USA) software package was used to analyse the data at 5% significance. Supplementary Table S6 shows the three experimental factors and levels chosen for this experiment. For the three factors involved, the second-order model equation used is as in Eq. (3):

3 $$ Y \, = X_{0} + X_{a} A + X_{b} B \, + X_{c} C \, + X_{ab} AB + X_{ac} AC \, + X_{bc} BC \, + X_{aa} A^{2} + \, X_{bb} B^{2} + X_{cc} C^{2} $$

*Y* = predicted response; *X*_*0*_ = intercept; *X*_*a*_, *X*_*b*_, *X*_*c*_ = coefficient estimates for the linear terms; *X*_*ab*_, *X*_*ac*_, *X*_*bc*_ = coefficient estimates for the interaction terms; *X*_*aa*_, *X*_*bb*_, *X*_*cc*_ = coefficient estimates for the quadratic terms; *A* = Co^2+^concentration, *B* = pH and *C* = Reaction time.

### Interference analysis

The selectivity of TLA towards Co^2+^ in the presence of other metal ions (Fe^3+^, Ni^2+^, Cu^2+^, Zn^2+^, Hg^2+^, Ag^+^, Pb^2+^, K^+^, Na^+^, Mg^2+^, Mn^2+^, Al^3+^, Cr^3+^, Ba^2+^, Ca^2+^, La^3+^) was studied via an interference analysis^25^. A control solution using the optimum result from the RSM model was applied, and its selectivity towards other metal ions at 1 and 5 equivalents were analysed.

### Sensitivity, LOD and job’s plot

The sensitivity of TLA towards Co^2+^ was studied by mixing 100 µM TLA with various concentrations of Co^2+^. The calibration curve for absorbance at the selected wavelength was constructed. The stoichiometry of the TLA-Co^2+^ interaction was investigated via the Job’s plot approach^26^.

### COSMO-RS

The geometries and the continuum solvation COSMO calculations of molecular surface density of the solvent and the titled compound were optimized and obtained using DFT calculations with BeckeePerdew-86 (BP86) functional and triple zeta valence potential (TZVP) basis set. The COSMO-files containing the ideal screening charges on the molecular surface were generated and used for the generation of sigma profile and polarity of the titled compounds^21^. All the above quantum calculations were carried out using the Amsterdam Density Functional (ADF) package, version 2017.

### DFT

The DFT calculation was conducted with the Gaussian09, Revision A.02 program. The B3LYP/6–311 +  + G(d,p) and CAM-B3LYP/LANL2DZ levels were used for calculating the chemical properties of TLA and TLA-Co^2+^, respectively. The molecular geometry optimization, HOMO and LUMO electron distributions were constructed using the Gauss View 5.0 program. The TD-DFT study used the CAM-B3LYP/LANL2DZ and IEF-PCM methods to calculate the electronic transition of TLA-Co^2+^ in DMSO solvent. The transition energies were predicted by calculating the first 50-singlet excited state.

### Applications for environmental water samples

The sensing ability of TLA was further tested using different environmental water samples such as distilled, tap, lake water, mangrove, and sea water that were spiked with Co^2+^. All water samples were centrifuged at 3500 rpm for 10 min, filtered and treated with UV light (254 nm) for 2 h. Solutions containing the optimized condition were prepared. For the test strip experiment, cellulose-based papers (1 × 5 cm^2^) were prepared by immersing them in the optimized solution, then oven-drying them. The dry test strips were then dipped in the spiked environmental water samples.

## Supplementary Information

Supplementary Information.
