# Supplementary material for: A highly sensitive and selective thiosemicarbazone chemosensor for detection of Co2+ in aqueous environments using RSM and TD/DFT approaches
Source: Sci Rep. 2021 Oct 25;11:20963. doi: 10.1038/s41598-021-00264-z (PMC8545950; doi:10.1038/s41598-021-00264-z)
Supplement: Supplementary file 1 — Supplementary Information. [file 41598_2021_264_MOESM1_ESM.docx]

**Supplementary Information**

*for*

**A highly sensitive and selective thiosemicarbazone chemosensor for detection of Co^2+^ in aqueous environments using**

**RSM and TD/DFT Approaches**

Hakimah Ismail^1^, Mohammad Norazmi Ahmad^1,2,3,4^ & Erna Normaya^1,2,3 *^

^1^Experimental and Theoretical Research Laboratory, Department of Chemistry, Kulliyyah of Science, International Islamic University Malaysia, Jalan Sultan Haji Ahmad Shah, Bandar Indera Mahkota, 25200, Kuantan, Pahang, Malaysia.

^2^River of Life Kuantan Chapter, International Islamic University Malaysia, Jalan Sultan Haji Ahmad Shah, Bandar Indera Mahkota, 25200, Kuantan, Pahang, Malaysia.

^3^Innovative Toyyib Environmental Minds, International Islamic University Malaysia, Jalan Sultan Haji Ahmad Shah, Bandar Indera Mahkota, 25200, Kuantan, Pahang, Malaysia.

^4^Drug and Poison Call Centre, IIUM Poison Centre, Office of Campus Director, International Islamic University Malaysia, Jalan Sultan Haji Ahmad Shah, Bandar Indera Mahkota, 25200, Kuantan, Pahang, Malaysia.

*email: ernanormaya@gmail.com

| **Std** | **Run** | ***A*: Co^2+^ concentration (μm)** | ***B*: pH** | ***C*: Time (minutes)** | **Absorbance at 450 nm** | **Standard deviation** |
| --- | --- | --- | --- | --- | --- | --- |
| 7 | 3 | 40 | 7.5 | 20 | 0.1691 | 0.0023 |
| 3 | 6 | 40 | 7.5 | 10 | 0.1939 | 0.0008 |
| 12 | 5 | 60 | 7.5 | 15 | 0.2406 | 0.0043 |
| 8 | 15 | 80 | 7.5 | 20 | 0.2629 | 0.0023 |
| 9 | 9 | 40 | 6.0 | 15 | 0.2670 | 0.0016 |
| 4 | 4 | 80 | 7.5 | 10 | 0.2697 | 0.0066 |
| 5 | 19 | 40 | 4.5 | 20 | 0.2702 | 0.0109 |
| 1 | 16 | 40 | 4.5 | 10 | 0.2798 | 0.0023 |
| 14 | 8 | 60 | 6.0 | 20 | 0.3208 | 0.0018 |
| 17 | 14 | 60 | 6.0 | 15 | 0.3214 | 0.0013 |
| 19 | 12 | 60 | 6.0 | 15 | 0.3245 | 0.0021 |
| 18 | 1 | 60 | 6.0 | 15 | 0.3246 | 0.0004 |
| 15 | 10 | 60 | 6.0 | 15 | 0.3255 | 0.0003 |
| 16 | 11 | 60 | 6.0 | 15 | 0.3255 | 0.0081 |
| 20 | 7 | 60 | 6.0 | 15 | 0.3273 | 0.0008 |
| 13 | 17 | 60 | 6.0 | 10 | 0.3317 | 0.0009 |
| 11 | 18 | 60 | 4.5 | 15 | 0.3365 | 0.0084 |
| 10 | 20 | 80 | 6.0 | 15 | 0.3379 | 0.0078 |
| 2 | 2 | 80 | 4.5 | 10 | 0.3479 | 0.0024 |
| 6 | 13 | 80 | 4.5 | 20 | 0.3501 | 0.0007 |

**Table S1.** RSM experiments with absorbance values at 450 nm.

| **Source** | **Sum of squares** | **df^a^** | **Mean square** | ***F*-value** | ***p*-Value** | ***t*-value** |
| --- | --- | --- | --- | --- | --- | --- |
| Model | 4.8975 × 10^-2^ | 9 | 5.4417 × 10^-3^ | 701.89 | < 0.0001 |  |
| *A* | 1.5093 × 10^-2^ | 1 | 1.5093 × 10^-2^ | 1946.78 | < 0.0001 | 44.12 |
| *B* | 2.0097 × 10^-2^ | 1 | 2.0097 × 10^-2^ | 2592.22 | < 0.0001 | -50.91 |
| *C* | 2.4900 × 10^-4^ | 1 | 2.4900 × 10^-4^ | 32.12 | 0.0002 | -5.67 |
| *AB* | 5.8320 × 10^-5^ | 1 | 5.8320 × 10^-5^ | 7.52 | 0.0207 | 2.74 |
| *AC* | 1.1101 × 10^-4^ | 1 | 1.1101 × 10^-4^ | 14.32 | 0.0036 | 3.78 |
| *BC* | 7.3205 × 10^-5^ | 1 | 7.3205 × 10^-5^ | 9.44 | 0.0118 | -3.07 |
| *A*^2^ | 1.3603 × 10^-3^ | 1 | 1.3603 × 10^-3^ | 175.46 | < 0.0001 | -13.25 |
| *B*^2^ | 3.5920 × 10^-3^ | 1 | 3.5920 × 10^-3^ | 463.30 | < 0.0001 | -21.52 |
| *C*^2^ | 6.6846 × 10^-6^ | 1 | 6.6846 × 10^-6^ | 0.86 | 0.3750 | 0.93 |
| Residual | 7.7529 × 10^-5^ | 10 | 7.7529 × 10^-6^ |  |  |  |
| Lack of Fit | 5.8609 × 10^-5^ | 5 | 1.1722 × 10^-5^ | 3.10 | 0.1201 |  |
| Pure Error | 1.8920 × 10^-5^ | 5 | 3.7840 × 10^-6^ |  |  |  |
| Cor Total^b^ | 4.9053 × 10^-2^ | 19 |  |  |  |  |

^a^ Degrees of freedom.

^b^ Total for all information corrected for the mean.

**Table S2.** ANOVA results for TLA-Co^2+^.

| **Terms** | **Value** |
| --- | --- |
| Standard deviation | 0.0028 |
| Mean | 0.2963 |
| Coefficient of variation (CV) % | 0.9396 |
| *R*^2^ | 0.9984 |
| Adjusted *R*^2^ | 0.9970 |
| Predicted *R*^2^ | 0.9887 |
| Adequate precision | 91.8596 |
| PRESS | 0.0006 |

**Table S3.** Fit statistics for the model.

| **Atom** | ***f*_k_^–^** | **Atom** | ***f*_k_^–^** |
| --- | --- | --- | --- |
| 1C | 0.010773 | 12C | 0.012063 |
| 2C | 0.013121 | 13N | 0.011231 |
| 3N | 0.012267 | 14H | 0.001358 |
| 4N | 0.022359 | 15H | 0.000492 |
| 5C | 0.029645 | 16H | 0.000693 |
| **6S** | **0.730495** | 17H | 0.000734 |
| 7N | 0.012323 | 18H | 0.000090 |
| 8C | 0.004801 | 19H | 0.011151 |
| 9C | 0.001904 | 20H | 0.011043 |
| **10N** | **0.161610** | 21H | 0.007643 |
| 11C | 0.002477 | 22H | 0.007534 |

**Table S4.** Values of the condensed electrophilic Fukui function for TLA.

| **Experimental** | | **Calculated** | | **Major contribution (≤ 25%)** | **Character** |
| --- | --- | --- | --- | --- | --- |
| **λ_max_ (nm)** | **Excitation energy (eV)** | **λ_max_ (nm)** | **Excitation energy (eV)** |  |  |
| 326 | 3.8032 | 310 | 3.9995 | 35% (HOMO-11 → LUMO) | n - π* + π - π * |
|  |  |  |  | 28% (HOMO-11 → LUMO+4) | n - π* + π - π* |
|  |  |  |  | 26% (HOMO-7 → LUMO+4) | n - π* |
| 383 | 3.2372 | 362 | 3.4250 | 91% (HOMO-3 → LUMO+1) | MLCT |
| 450 | 2.7552 | 457 | 2.7130 | 95.50% (HOMO → LUMO+7) | MLCT + LMCT |

**Table S5.** Experimental and calculated electronic transitions, excitation energies and the major contributions and their characters for TLA-Co^2+^.

| **Factors** | **Unit** | **Notation** | **Variable levels** | | |
| --- | --- | --- | --- | --- | --- |
|  |  |  | **– 1** | **0** | **+ 1** |
| Co^2+^concentration | μM | *A* | 40 | 60 | 80 |
| pH | - | *B* | 4.5 | 6.0 | 7.5 |
| Reaction time | Minutes | *C* | 10 | 15 | 20 |

**Table S6.** Experimental factors and levels used in the RSM experiment.


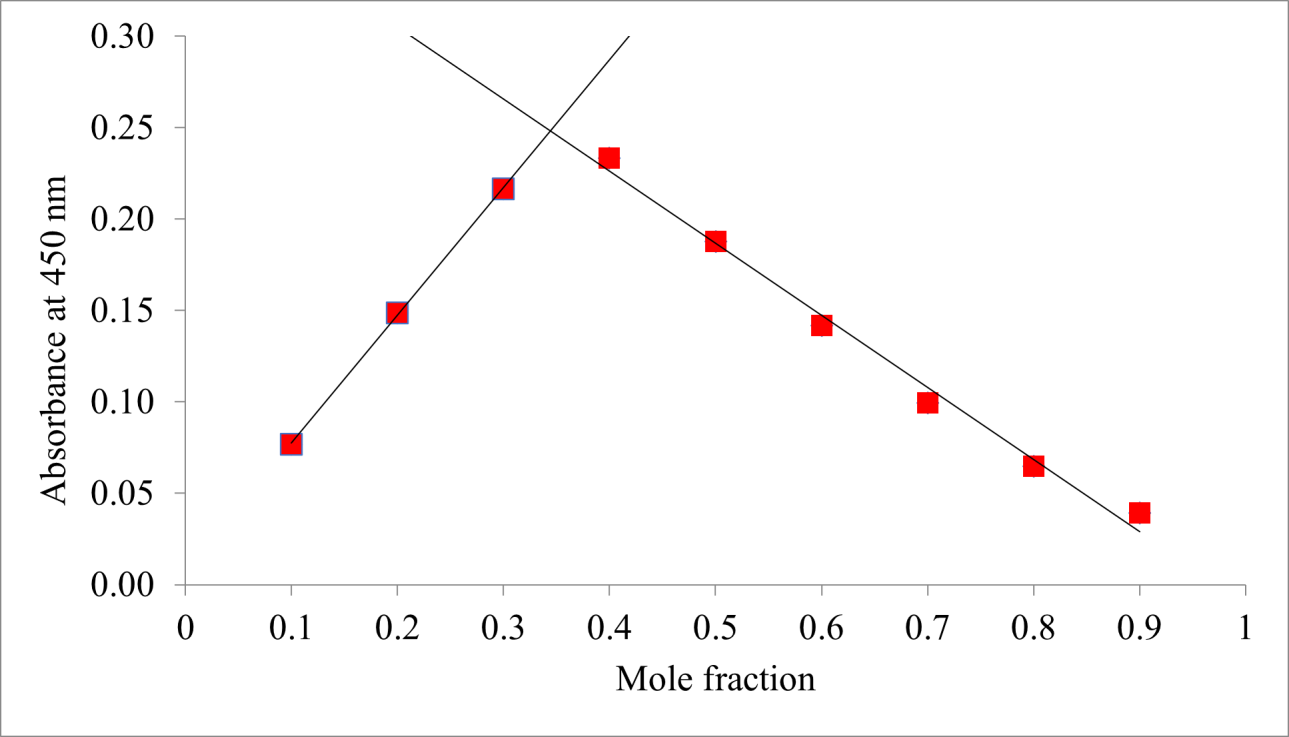


**Figure S1.** Job’s plot for the determination of the stoichiometry of TLA and Co^2+^ in the complex monitored at 450 nm.


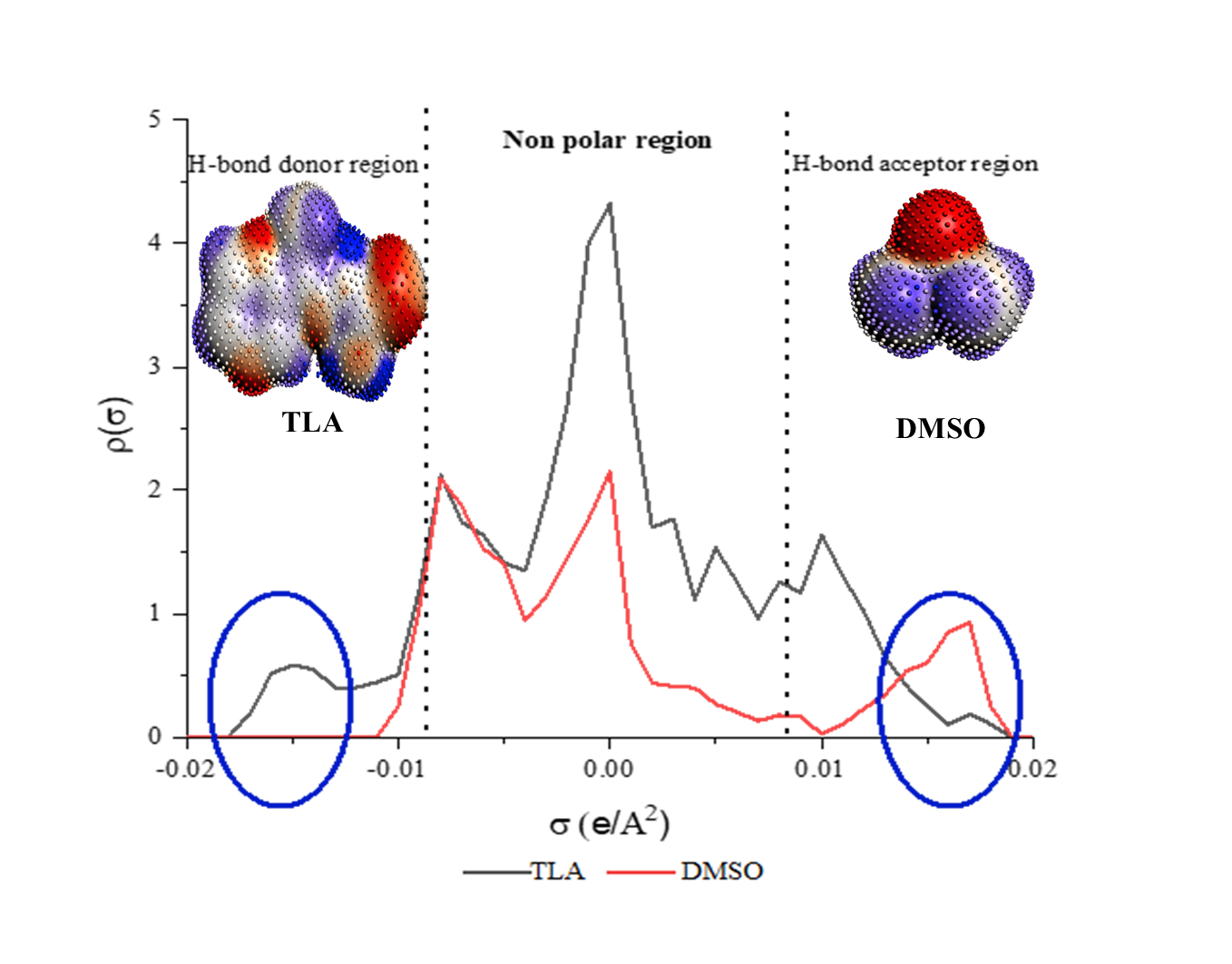


**Figure S2.** Sigma profiles of TLA and DMSO.





**Figure S3.** Correlation coefficient between experimental and optimized structure.


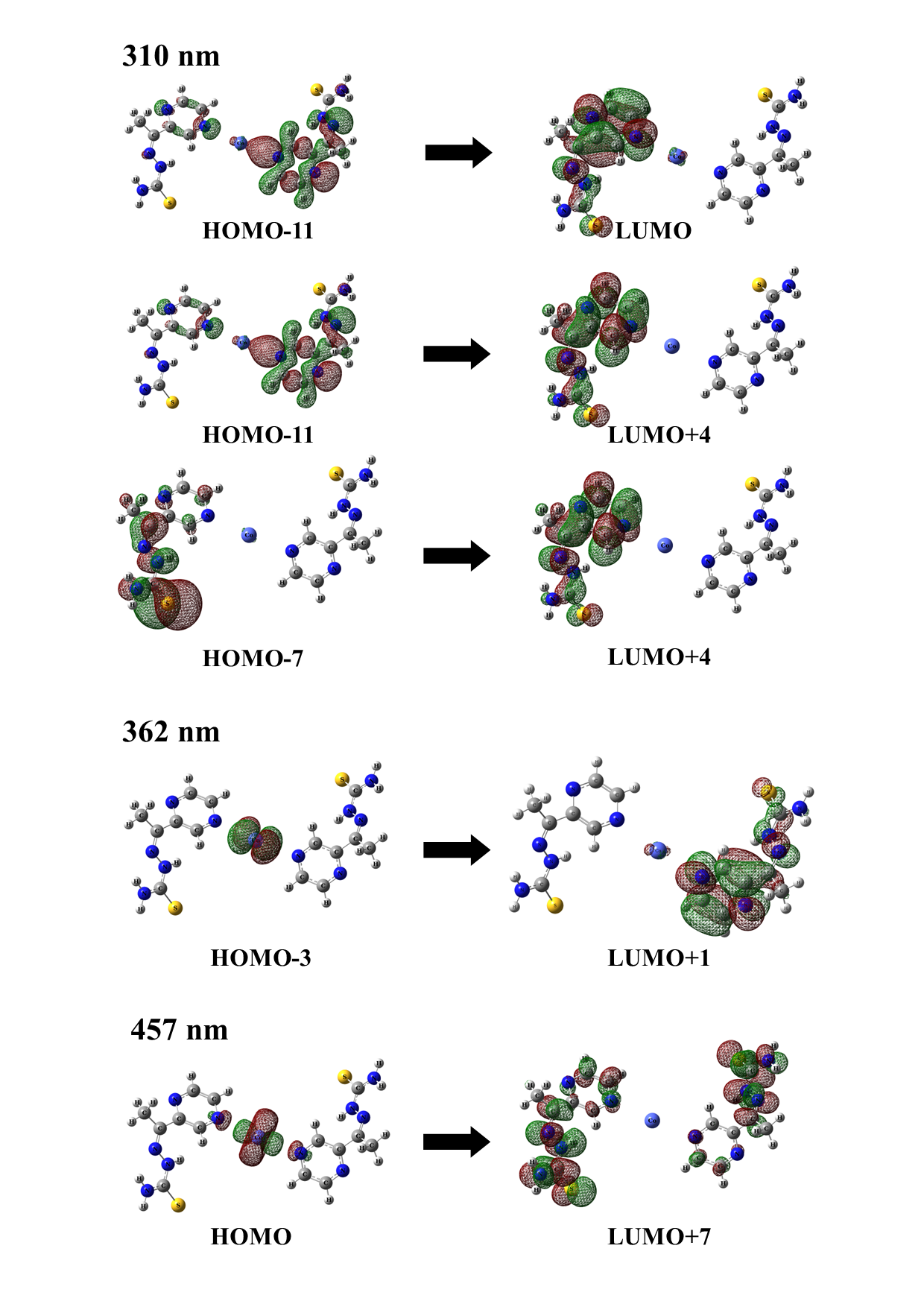


**Figure S4.** Contour plot overview of TLA-Co^2+^.


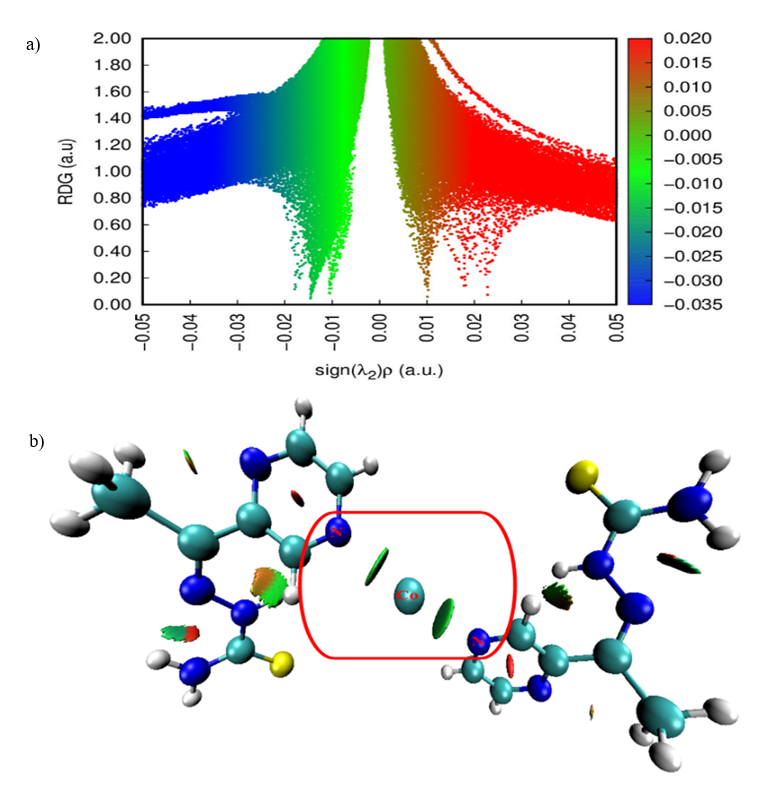


**Figure S5.** (a) NCI-RDG scatter diagrams of TLA-Co^2+^; (b) Colour-mapped RDG isosurface graph.


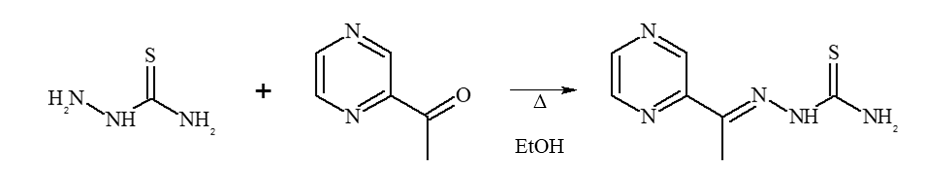


**Figure S6.** The synthetic route of TLA.


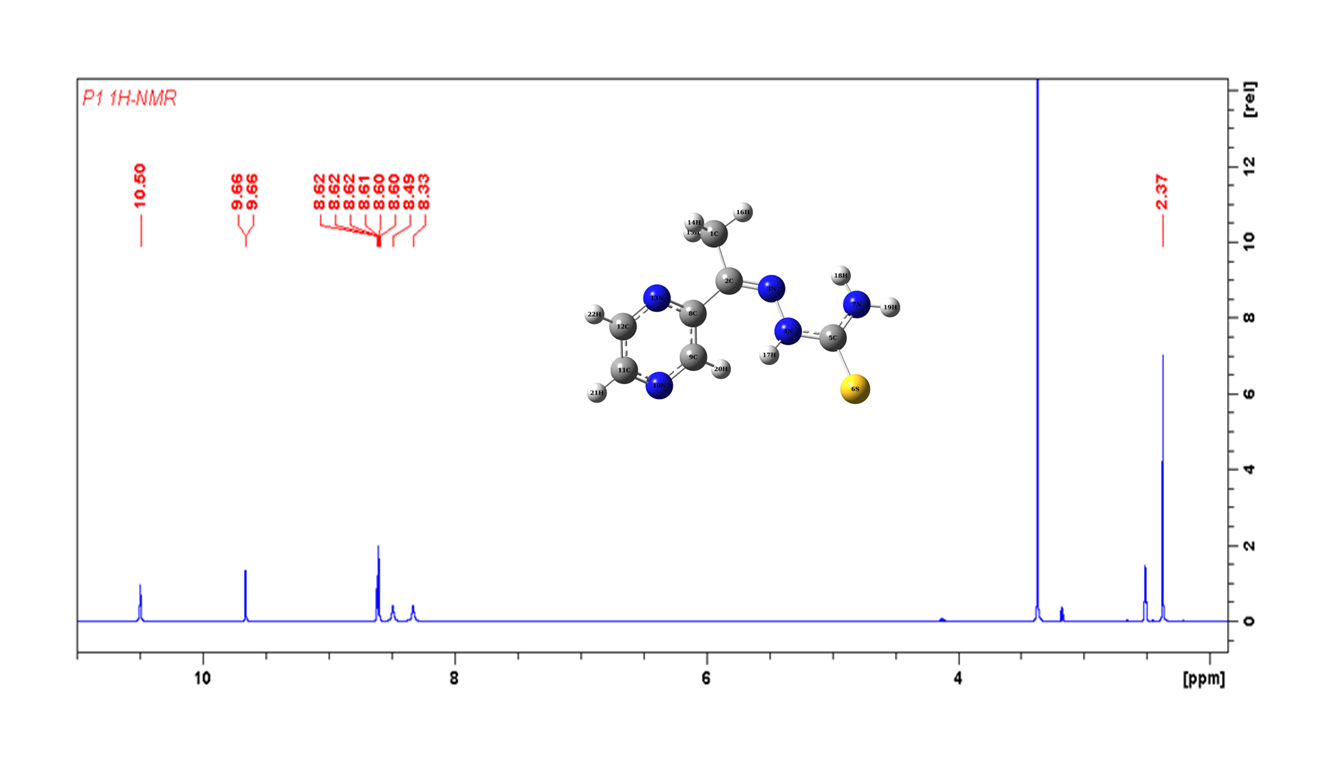


**Figure S7**. ^1^H-NMR spectrum of TLA


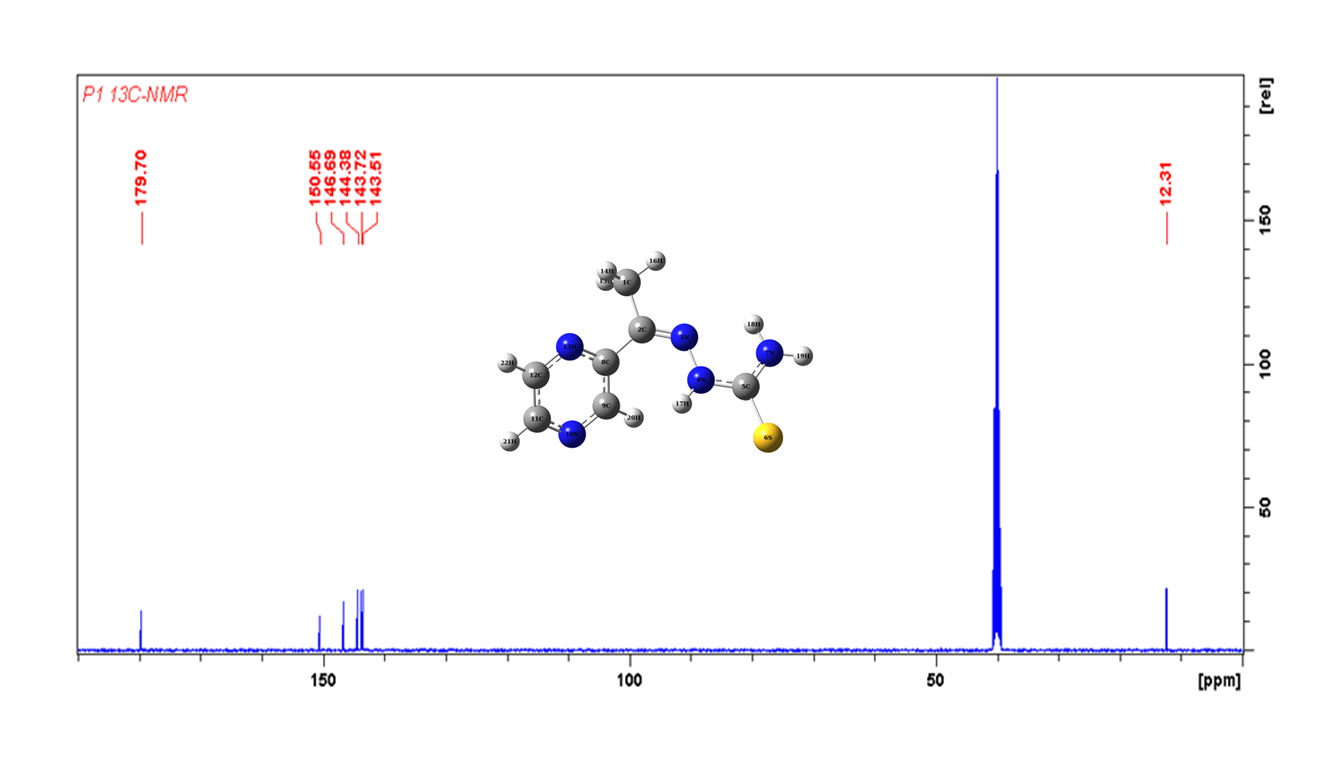


**Figure S8.** ^13^C-NMR spectrum of TLA


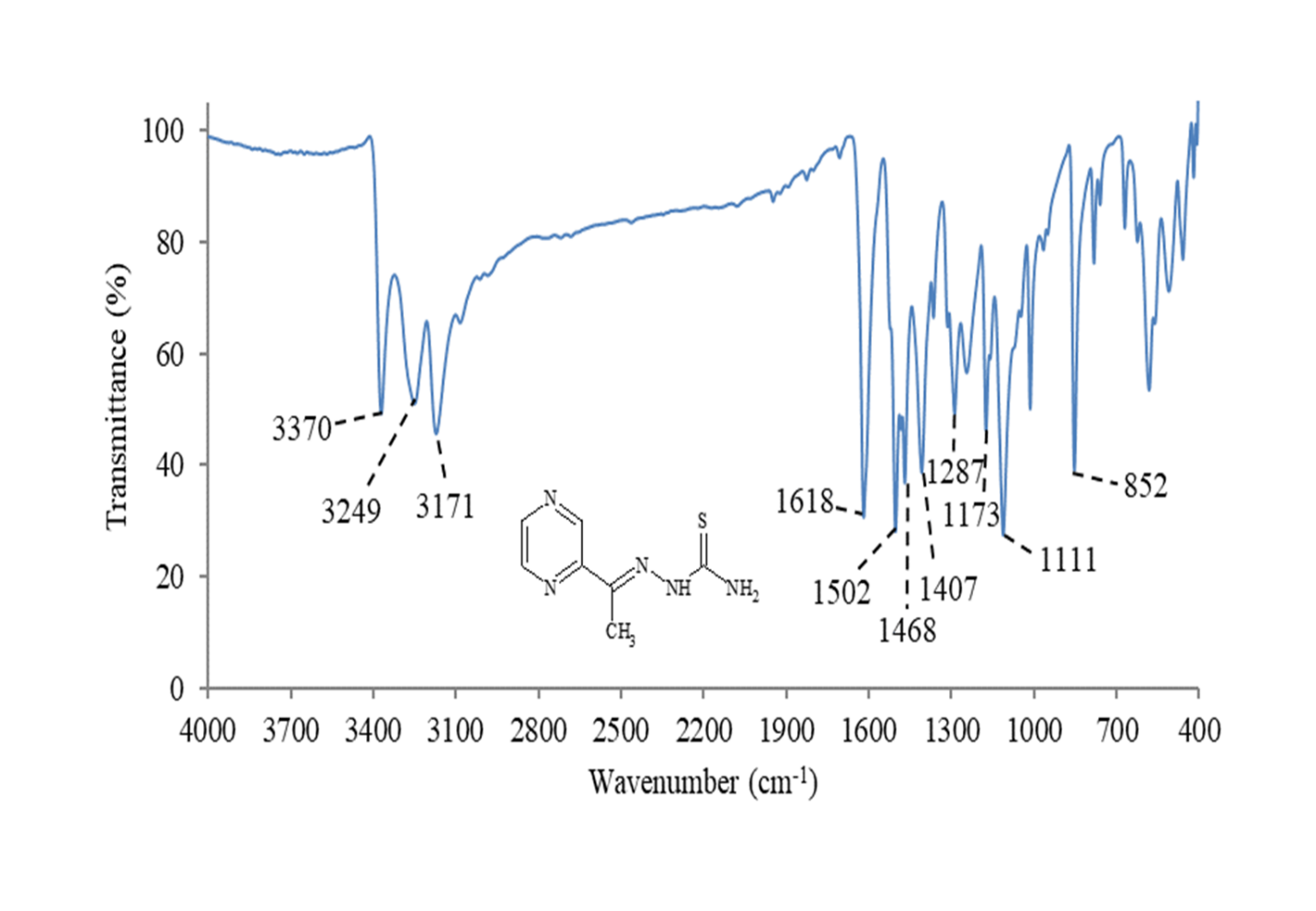


**Figure S9**. Infrared spectrum of TLA
